# Study of Expression of P16 in Premalignant and Malignant Lesions of Penis and Their Significance

**DOI:** 10.30699/IJP.2024.1998898.3092

**Published:** 2024-03-29

**Authors:** Manish Shetty, Deepa Sowkur Anandarama Adiga, Chaithra G V

**Affiliations:** *Department of Pathology, Kasturba Medical College, Mangalore, Manipal Academy of Higher Education, Manipal, Karnataka, India*

**Keywords:** India, Immunohistochemical staining, Oncogenic HPV, p16, Penile SCC, SCC

## Abstract

**Background & Objective:**

Penile squamous cell carcinoma (SCC) is an extremely rare malignancy. It is usually caused by chronic human papillomavirus (HPV) 16 and HPV 18 infections. This study was conducted to investigate the immunohistochemical overexpression of p16, a surrogate marker for HPV, and to evaluate its usefulness as a potential diagnostic biomarker.

**Methods:**

In this cross-sectional prospective and retrospective cohort study, 56 penile squamous cell carcinoma (SCC) specimens and five penile premalignant specimens were evaluated in Kasturba Medical College, Mangalore, India, from January 2013- December 2018 in terms of clinical and histopathological features. Immunohistochemical expression for p16 in cases and controls was evaluated. Statistical comparison of p16 expression among clinical features, histological subtype, grade, and stages of tumor were done.

**Results:**

Analysis of the pattern of p16 staining showed diffuse and strong nuclear and cytoplasmic expression in 32.8% of the cases. There was a highly significant association (*P*<0.001) of pattern of p16 expression among the HPV and non-HPV subtypes of penile carcinoma. p16 expression was not significantly associated with other prognostic parameters like site of the lesion, lymphovascular invasion, perineural invasion, histologic grade, and pathologic stage.

**Conclusion:**

Expression of p16 would be a useful tool in differentiation between the HPV-associated and non-HPV-associated subtypes of penile SCC that may be helpful in prediction of aggressiveness and invasive potential of the respective histologic subtypes.

## Introduction

Penile cancer is a rare malignancy, accounting for around 0.84 cases per 100,000 persons (1). The incidence is much higher in South America, Asian and African countries, compared to global incidence (1, 2). Squamous cell carcinoma (SCC) is the most common histological type of penile cancer, which is responsible for 95% of the cases (2,3). 

The various etiologies include phimosis, poor hygiene, smoking, and chronic inflammatory states. Lesions like Bowen's disease and Bowenoid Papulosis, the forms of carcinoma in situ, have known associations with human papillomavirus (HPV). In particular, infection with HPV has been linked to penile cancer carcinogenesis (4, 5, 6). 

 Common high-risk HPV types are HPV16 and HPV18, and they exert their oncogenic effects by expressing the E6 and E7 oncoproteins, which bind and inactivate p53 and pRB proteins. The oncogenic HPVs interfere with the cell cycle control, affecting both cell multiplication and apoptosis, which causes functional dysregulation of pRB by E7 of the HPV resulting in the reciprocal overexpression of p16INK4A (7).

 p16 protein (p16) is a cyclin-dependent kinase (CDK) inhibitor that regulates cell cycle by inactivating the CDKs that phosphorylate retinoblastoma (Rb) protein. Other studies have also revealed that the status of Rb expression markedly influences p16 expression, and p16 overexpression has been demonstrated in genital cancers because of functional inactivation of Rb by HPV E7 protein (8).

HPV status in tumors can be assessed by several techniques, namely HPV DNA detection by in situ hybridization, PCR, and quantitative reverse transcriptase to detect HPVE6/E7RNA expression (9).

Recent studies have shown that overexpression of p16 using immunohistochemical staining can be used as a surrogate marker for high-risk HPV-induced penile carcinomas (10, 11, 12, 13, 14). HPV-induced cancers are considered when more than 70% of cytoplasmic and nuclear staining is seen in the tumoral tissue (13).

 Although molecular methods are considered as the gold standard, the advantages of p16 immunohistochemical analysis, namely simplicity, low cost, and high sensitivity, have brought the placing of more profound methods, such as PCR-based and HPV DNA in situ hybridization, to a rarer end. However, the drawbacks and concerns brought about by this routine diagnostic approach are an inclination toward false-positive results due to variations in the technique and reporting of staining and a lack of evidence of the association between the integration of HPV DNA and the expression of p16. (15, 16)

Histologically, penile squamous cell carcinoma is classified broadly into non-HPV-associated ones and HPV-associated ones. The non-HPV associated subtypes include the usual type, pseudohyperplastic, pseudoglandular, verrucous, papillary, carcinoma cuniculatum, adenosquamous, sarcomatoid, and mixed subtypes. The HPV-associated subtypes included basaloid, papillary basaloid, warty, warty basaloid, clear cell, and lymphoepithelioma-like SCC (6). Recent evidence suggests that p16 positivity is associated with a lower risk of death and improved survival (16). 

Despite its unpredictable behavior and aggressive treatment, there have only been a few reports regarding epigenetic mechanisms in the development of penile carcinoma. Epigenetic alterations of the genes, such as expression of p16, may aid in identifying penile carcinoma and its premalignant lesions, revealing candidates for development of specific markers for cancer detection, diagnosis, and prognosis (17).

Thus, this study was conducted as a prospective study to ascertain overexpression of p16 through immunohistochemical staining in premalignant and malignant lesions of the penis and to evaluate its usefulness as a potential diagnostic biomarker.

## Material and Methods

**Study Subjects**

In this retrospective and prospective cross-sectional study, all cases of penile squamous cell carcinoma and penile intraepithelial neoplastic (PeIN) lesions that included excision biopsy were evaluated in Kasturba Medical College, Mangalore, a tertiary health care sector catering to a large population of coastal Karnataka. The study duration was from January 2013 to December 2018. All cases with inadequate tissue content or improperly processed tissue were excluded. Thirty-six inflammatory conditions of the penis, including chronic balanoposthitis and phimosis due to non-neoplastic causes, were used as negative controls for p16 IHC.

The computerized database of the institution was searched for patients with premalignant and malignant lesions of the penis to obtain the clinical details of all the patients. Histopathological examination findings included each case's histological subtype, grading, and pathologic staging. The Institutional Ethics Committee of Kasturba Medical College, Mangalore, approved the study.

**Histological Evaluation**

Firstly, the Hematoxylin and Eosin stained slides for each case were examined for histologic features, including subtypes, grade, pathologic stage (as per AJCC, 8^th^ ed 2017), presence or absence of perineural and lymphovascular invasion, and lymph node metastasis.

**Immunohistochemistry for p16**

Immunohistochemical staining for p16 was done on slides prepared from biopsies or resection specimens obtained from each case, and the findings were recorded and evaluated for the presence or absence of p16 staining.

**Procedure for p16 Immunostaining**

Paraffin-embedded tissue sections were applied on poly-L-Lysine coated slides and kept overnight at 370°C, followed by de-paraffinization using xylene and then dehydrated through graded alcohol. The slides were then treated with hydrogen peroxide in methanol, following which antigen retrieval by microwave irradiation for 8 minutes and washing with working solution (0.1M Tris-HCl, 0.15M NaCl, pH 7.4) was done. The slides were then incubated with primary antibody anti p16(INK4) for 30 minutes at room temperature, after which treatment with Tris buffer for 10 minutes was done. Diaminobenzidene chromogen was added, and counterstaining with Meyer's hematoxylin was done. Then, the washed slides were dried and mounted with dibutyl phthalate polystyrene xylene.

The controls were assessed concurrently with the test slides.

**Fig. 1:**
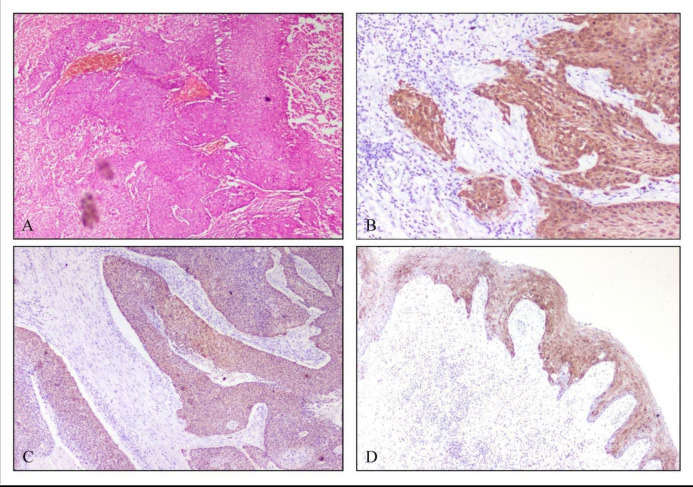
Photomicrograph showing papillary basaloid squamous cell carcinoma H&E 200X **(A).** Strong and diffuse cytoplasmic and nuclear positivity for p16 in warty basaloid variant, (**B**). Papillary basaloid variant (**C),** and Condyloma accuminata (**D**)**.**

Immunohistochemical staining for p16 was assessed for 61 cases involved in the study. The presence of both nuclear and cytoplasmic staining was taken as positive (Figure 1). The analysis of p16 expression was based on the expression pattern and classified into diffuse and strong (more than 70% tumor cells showing nuclear and cytoplasmic positivity) and negative cases.

**Statistical Analysis**

Comparison of p16 expression between age, histological subtype, grade, stage, and tumor site was done using software SPSS version 23 (SPSS Inc., Chicago, Ill., USA). The collected data was analyzed with frequency and percentage. The correlations between p16 expression and other parameters were tested for significance using the Chi-Square/Fisher's exact test. The data was considered significant with a P-value<0.05 and highly significant with a P-value<0.001.

## Results

The study involved a total of 97 samples comprising of 61 cases and 36 controls. Among the cases diagnosed during the period January 2013 to December 2018, 56 were malignant and 5 were premalignant. All the malignant cases were squamous cell carcinoma. The premalignant lesions included two cases of balanitis xerotica obliterans, two cases of Condyloma acuminatum, and one of Bowen's disease. 

The mean age of the cases was 57.25±13.02 years. The majority comprised twenty-six (42.6%) patients had a history of phimosis, whereas 12 (19.7%) patients did not have phimosis, and the data for the rest (37.7%) of the patients were unavailable. Also, p16 expression was not significantly associated with a history of phimosis (*P*=0.101). The clinical characteristics of the patients are shown in Table 1.

**Table 1:** Clinical characteristics

Clinical Details		Groups			
		Cases		Controls	
		Count	%	Count	%	
Age	21 – 40	6	9.8	18	50.0	
	41 – 60	31	50.8	8	22.2	
	61 – 80	22	36.1	9	25.0	
	Above 80	2	3.3	1	2.8	
	Total	61	100.0	36	100.0	
Phimosis	Yes	26	42.6	28	77.8	
	No	12	19.7	8	22.2	
	Unavailable	23	37.7	0	0	
	Total (Including Unavailable history)	61	100.0	36	100.0	
Diabetes Mellitus	Unknown	38	62.2	16	44.4	
	Present	3	4.9	9	25.0	
	Absent	20	32.7	11	30.6	
	Total	61	100.0	36	100.0	
Hypertension	Unknown	38	62.3	16	44.4	
					
	Present	8	13.1	18	50.0	
	Absent	15	24.6	2	5.6	
	Total	61	100.0	36	100.0	

Out of the 48 cases where the tumor location in the penis was known, the majority were localized to the glans penis. Histopathological examination findings are presented in Table 2. 

Most of the tumors were of the SCC 'Usual' histologic subtype, accounting for 17 cases (27.9%). The least common subtype was the pseudoglandular variant, which accounted for 1 case. Most were low-grade tumors (G1), well-differentiated (57.4%). Most tumor samples belonged to pTNM stage 2 (50.8%). Lymphovascular invasion was present in 6/61 of the cases, and perineural invasion was present in 10/61 of the tumor samples. p16 expression was not significantly associated with other prognostic parameters like site of the lesion (*P*=0.108), lymphovascular invasion (*P*=0.344), perineural invasion (*P*=0.045), histologic grade (*P*=0.068) and pathologic staging (*P*=0.210) as shown in Table 2.

**Table 2:** Histopathological Data

Anatomical Localization	Frequency(n=61)	Percentage	Association with p16^(Pearson Chi-square test)^
Glans penis	36	59.0	0.108
Prepuce	6	9.8	
Both Glans and Prepuce	6	9.8	
Unknown	13	21.4	
Total(Including unknown)	61	100	
**Histologic Subtype**	**Frequency (n=61)**	**Percentage**	
Usual type	17	27.9	0.00
Verrucous	12	19.7	
Warty basaloid	6	9.8	
Warty	7	11.5	
Papillary	7	11.5	
Papillary basaloid	2	3.3	
Low grade PeIN	5	8.1	
Pseudohyperplastic	2	3.3	
Pseudoglandular	1	1.6	
Carcinoma cuniculatum	2	3.3	
Histological Grade	**Frequency (n=61)**	**Percentage**	
Grade 1	35	57.4	0.068
Grade 2	18	29.5	
Grade 3	3	4.9	
Unknown	5	8.2	
Stage	**Frequency(n=61)**	**Percentage**	
Stage 1	14	22.3	0.210
Stage 2	31	50.8	
Stage 3	7	11.5	
Stage 4	4	6.6	
Unknown	5	8.2	
Lymphovascular Invasion	**Frequency (n=61)**	**Percentage**	
Present	6	9.8	0.344
Absent	55	90.2	
Perineural Invasion	**Frequency (n=61)**	**Percentage**	
Present	10	16.4	0.045
Absent	51	83.6	

According to the World Health Organization (WHO), classification of squamous cell carcinoma (6), the histologic subtypes were further grouped into non-HPV-associated and HPV associated types, as mentioned before, to study the statistical significance of the expression of p16. This classification of the subtypes is represented below in Table 3

**Table 3:** Classification of the HPV/Non-HPV associated Subtypes

	Subtypes	Frequency(n=61)
HPV-associated Subtypes (n=20)	Low Grade PeIN(warty)	5
	Papillary Basaloid	2
	Warty	7
	Warty basaloid	6
Non-HPV-associated Subtypes (n=41)	Carcinoma cuniculatum	2
	Papillary	7
	Pseudoglandular	1
	Pseudohyperplastic	2
	Usual type	17
	Verrucous	12

The cases showed primarily two types of expression patterns for the p16 immunostaining, as shown in Figure 1. Hence, the p16 staining pattern was classified into two groups, diffuse and strong and negative, based on the cytoplasmic and nuclear response to the stain. 

**Fig. 2:**
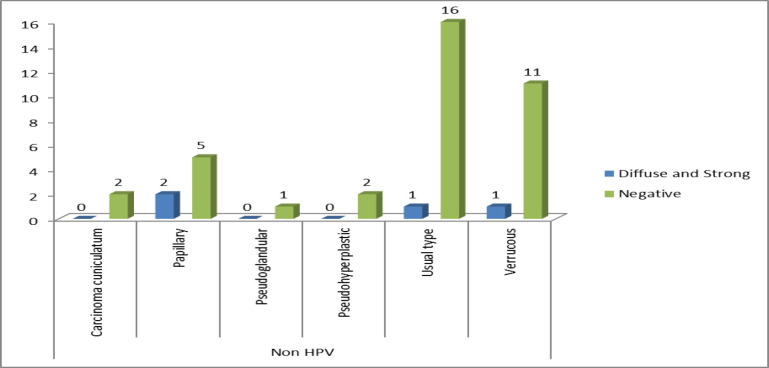
Chart depicting the p16 expression in non- HPV associated subtypes of penile SCC

The expression patterns for the p16 immunostaining shown by each subtype of penile squamous cell carcinoma are depicted in Figures 1 and 2.

**Fig. 3:**
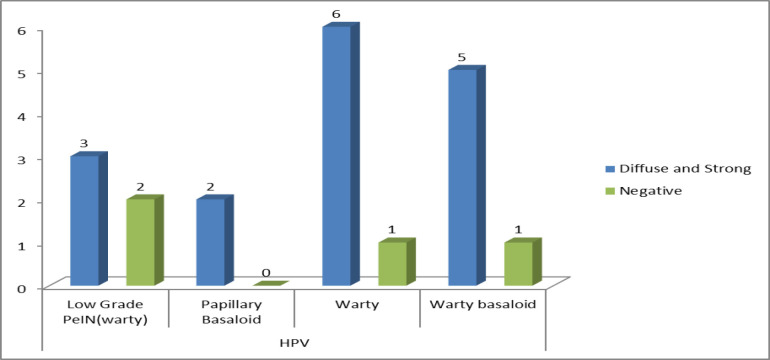
Chart showing p16 expression in the HPV-associated subtypes of penile SCC

In accordance with the WHO classification of each histologic subtype into either HPV-associated or non-HPV-associated (6), the data was correlated with the p16 expression. There was found to be a highly significant association (<0.001) between the p16 expression patterns and the HPV or non-HPV subtypes of penile carcinoma. The majority of the HPV-associated subtypes showed a strong and diffuse pattern of expression, whereas, among non-HPV-associated subtypes, the majority showed an absence of p16, as seen in Table 4.

**Treatment Details, Follow-up, and Survival Data**

Regular follow-up dates as per the standard schedule were not available. One year of survival data by telephonic conversation could be obtained for 12 out of 56 cases of SCC. Four cases that belonged to stage 1 and stage 2 underwent circumcision and were cured.

**Table 4:** Correlation between p16 expression and HPV/non-HPV associated subtypes

**Histological Subtypes**	**p16 Expression**		**Total**	**P-value** (Pearson Chi-square test)
	Diffuse and Strong	Negative		
HPV-associated	16	4	20	<0.001 (HS)
Non-HPV-associated	4	37	41	

## Discussion

The frequent occurrence of penile carcinoma remains a health challenge, although less common in developed countries (18).

Penile squamous cell carcinoma accounted for all of the malignant lesions taken in our study. This included five PeINs along with 56 invasive types. This is comparable to a study where penile squamous cell carcinoma (PeSCC) accounted for 95% of the malignant lesions (7, 18). The most common anatomical site of the lesions was the glans penis in our study which is recorded as the most common site (6).

The risk factors for premalignant and malignant squamous lesions include presence of phimosis, HPV infection, and poor genital hygiene, as well as other penile lesions such as Bowen's disease, balanitis xerotica obliterans, and giant condyloma (4, 7). Our study found phimosis as the major risk factor (in twenty-six cases).

Age of the patients with PeSCC fell in the range of 31 to 86 years, which is similar to the age range shown in a study by Steinestel J* et al. *(19) and Shah AA* et al. *(18). The mean age of the cases was 57.25±13.02 years in our study, a finding seen in the compared study (18). The present study reported majority of the cases belong to well-differentiated (G1) grade of tumors and Stage 2 of TNM staging, which resembles the trend in the grade and stage of the study done by Do HTT *et al.* (22). The study found the usual subtype of PeSCC as the most common type followed by the verrucous type which is also comparable to the findings in our study.

Development of the penile squamous cell carcinoma and its association with high-risk HPV infection is evident in other studies. (1, 19, 21) In our study, we found that the number of non-HPV-associated cases predominated. This may be due to the less frequent prevalence of HPV infection, reflecting a better socioeconomic condition in this location. This also suggests the possibility of an alternate pathway for the pathogenesis of the carcinoma induced by other risk factors like phimosis. (18) 

Among the premalignant conditions, one of the cases was associated with adenocarcinoma of the lung. This association is probably due to smoking being the common etiological factor for both lung carcinoma and penile SCC. (4, 7)

Concerning the premalignant lesions of the penis, we analyzed five cases and found them to have a predominantly diffuse and strong p16 expression pattern. This is similar to a study performed on oral premalignant and malignant lesions infected with HPV. (22) This study found that a diffuse pattern of positivity was found mainly in premalignant and malignant lesions, which were HPV-positive. In contrast, the HPV-negative lesions had a sporadic or negative expression. It indicates a strong relation between the p16 protein's expression pattern and malignant lesions with high malignant potential. In our study, we found that majority (3/5) of the premalignant lesions classified as low-grade PeIN subtype, which were HPV associated, showed a diffuse and strong expression of p16. Accordingly, such HPV- associated PeIN is more likely to develop , as well as would be helpful to hypothesize that such HPV-associated PeINs are more likely to progress to basaloid and warty variants, while those associated with non-viral risk factors usually progress to a well-differentiated or keratinizing SCC (5).

Among the frankly malignant lesions, the p16 expression pattern in our study showed primarily a diffuse and strong pattern in HPV-associated subtypes and a negative expression in non-HPV-associated subtypes. This is comparable to a study done by Do HTT* et al. *(20) and Gregoire L* et al. *(22) with similar results. The study also analyzed the predisposing factors of PeSCC, such as the history of phimosis, which is the most common, akin to a frequency of risk factors similar to our study. majority of the cases gave a positive history, which is analogous to our study, where phimosis was one of the major complaints. (20) Moreover, the p16 expression was not significantly associated with stage, anatomical localization, and phimosis (*P*>0.05) in this study. Intense and nuclear staining had higher specificity with high-risk HPV. (19)

The association of p16 expression with each histologic subtype was not statistically significant in some studies (19,20). Contrastingly, in our study, the association between each histologic subtype and p16 expression was found to be highly significant (*P*<0.001). This result is similar to a study done by Martins VdA* et al., *where an association was observed between the subtype of tumor and p16 positivity, especially among HPV-associated subtypes such as the basaloid types (24).

However, in the current study, an element of sample bias cannot be excluded in this regard as each histologic subtype being evaluated contributed a small size for comparison.

The p16 expression with HPV-associated subtypes as a whole was found to be highly significant (*P*<0.001), which is in concordance with a study by Gregoire L* et al. *(23,25). Another study by Muresu N* et al. *(26) shares similar results where a statistically significant difference in p16 expression was present between HPV positive and negative cases. This statistical outcome between p16 and HPV/Non-HPV subtypes of oenile SCC could potentially advocate for using p16 Immunohistochemistry as a diagnostic tool in Penile SCC of the HPV-associated subtypes (26).

Furthermore, the HPV-associated subtypes had predominant basaloid morphology compared to non-HPV-associated types, predominantly keratinizing. This finding draws parallels to a recent study by Mohanty SK* et al. *(27) with similar demographics, where a strong association between the histological subtypes of the specimens and HPV positivity was noted (23, 28). 

Though the etiological role is well established, the importance of HPV as a prognostic factor in penile carcinoma has controversial results. Utilization of a combination of at least two diagnostic tests is warranted to improve diagnostic accuracy, albeit the association between p16 and HPV positive and negative cases was fair (19,25, 29, 30). 

In a meta-analysis study done in 2018 on men with penile cancer by Sand FL* et al. *(31), they highlighted the importance of p16 and HPV status as a prognostic factor, wherein they concluded that p16 or HPV-positive penile cancer had a greater disease-specific survival rate. They attributed this to an increased immune response due to the viral infection and a more suitable molecular profile. As such, they indicated that this expression could potentially be used as a predictive marker for better survival.

More recent studies also underline the potential of p16ink4a status as a prognostic factor in various other malignancies such as anal, oropharyngeal, vulvar, and vaginal as well as penile cancers. These studies also highlight that dual testing with both HPV and p16 can increase the prognostic capability in HPV-related cancers rather than just using one of them individually. (27,32, 33)

The prognostic value of p16 IHC could not be determined in our study as follow-up details were unavailable, which becomes a limitation of this study. Studies on the expression of p16 IHC and looking at association with long-term survival would determine the prognostic value.

## Conclusion

Apart from this data representative of the frequency of penile cancer in coastal Karnataka, India, the expression of p16 through immunohistochemical staining can be ascertained in our study in malignant and premalignant lesions of the penis. However, its role as a prognostic biomarker remains to be seen as recurrence and survival parameters were not considered in our study. It would be a useful tool in discriminating between the HPV-associated and non-HPV-associated subtypes of penile SCC to assess the aggressiveness and invasive potential of the respective histologic subtypes. Still, more studies would be required to ascertain the results.

## Funding

The Indian Council of Medical Research funded this project.

## Conflict of Interest

The authors declare no conflict of interest.
